# Hemoglobin Mass, Blood Volume and VO_2_max of Trained and Untrained Children and Adolescents Living at Different Altitudes

**DOI:** 10.3389/fphys.2022.892247

**Published:** 2022-06-03

**Authors:** Erica Mabel Mancera-Soto, Diana Marcela Ramos-Caballero, Joel A. Rojas J., Lohover Duque, Sandra Chaves-Gomez, Edgar Cristancho-Mejía, Walter Franz-Joachim Schmidt

**Affiliations:** ^1^ Departamento del Movimiento Corporal Humano, Facultad de Medicina, Universidad Nacional de Colombia, Bogotá, Colombia; ^2^ Department of Sports Medicine and Sports Physiology, University of Bayreuth, Bayreuth, Germany; ^3^ Departamento de Biología, Facultad de Ciencias, Universidad Nacional de Colombia, Bogotá, Colombia; ^4^ Escuela de Medicina y Ciencias de la Salud, Universidad del Rosario, Bogotá, Colombia; ^5^ Programa de Licenciatura en Educación Física Recreación y Deporte, Facultad de Ciencias de la Educación, Unidad Central del Valle del Cauca, Tuluá, Colombia; ^6^ Laboratorio de Control al Dopaje, Ministerio del Deporte de Colombia, Bogotá, Colombia

**Keywords:** puberty, tanner stage, erythropoiesis, hypoxia, lean body mass

## Abstract

**Introduction:** To a considerable extent, the magnitude of blood volume (BV) and hemoglobin mass (Hbmass) contribute to the maximum O_2_-uptake (VO_2_max), especially in endurance-trained athletes. However, the development of Hbmass and BV and their relationships with VO_2_max during childhood are unknown. The aim of the present cross-sectional study was to investigate Hbmass and BV and their relationships with VO_2_max in children and adolescents. In addition, the possible influence of endurance training and chronic hypoxia was evaluated.

**Methods:** A total of 475 differently trained children and adolescents (girls *n* = 217, boys *n* = 258; untrained *n* = 171, endurance trained *n* = 304) living at two different altitudes (∼1,000 m, *n* = 204, ∼2,600 m, *n* = 271) and 9–18 years old participated in the study. The stage of puberty was determined according to Tanner; Hbmass and BV were determined by CO rebreathing; and VO_2_max was determined by cycle ergometry and for runners on the treadmill.

**Results:** Before puberty, there was no association between training status and Hbmass or BV. During and after puberty, we found 7–10% higher values in the trained groups. Living at a moderate altitude had a uniformly positive effect of ∼7% on Hbmass in all groups and no effect on BV. The VO_2_max before, during and after puberty was strongly associated with training (pre/early puberty: boys +27%, girls +26%; mid puberty: +42% and +45%; late puberty: +43% and +47%) but not with altitude. The associated effects of training in the pre/early pubertal groups were independent of Hbmass and BV, while in the mid- and late pubertal groups, 25% of the training effect could be attributed to the elevated Hbmass.

**Conclusions:** The associated effects of training on Hbmass and BV, resulting in increased VO_2_max, can only be observed after the onset of puberty.

## Introduction

The maximum oxygen uptake (VO_2_max) of an adult athlete depends, among other factors, such as muscle fiber composition and cardiac compliance (Levine, 2008), to a large extent on the level of blood volume (BV) and hemoglobin since the BV determines the maximum cardiac output (CO), and the hemoglobin concentration ([Hb]) influences the maximum arterial-mixed venous oxygen difference ( $a-\bar{v}\;O_{2}\;\text{diff}$ ) (Lundby et al., 2017; Schierbauer et al., 2021). Therefore, the hemoglobin mass (Hbmass), as an integrative parameter of BV and [Hb], is closely associated with VO_2_max, and a change in Hbmass of 1 g is related to a change in VO_2_max of ∼4 ml min^−1^ (Schmidt and Prommer, 2010). Training in adulthood only causes a relatively small adjustment of the Hbmass by 3–6.5%, (Schmidt and Prommer, 2008; Garvican et al., 2010), so that a genetic predisposition or an early training adjustment in childhood and adolescence has been discussed to explain the high values in elite athletes exceeding those of untrained subjects by more than 40% (Heinicke et al., 2001). Findings from Martino et al., (2002) showed that completely untrained people with a VO_2_max of ∼65 ml kg^−1^ min^−1^ possess a significantly increased BV and a higher Hbmass, suggesting a genetic determination.

This work is part of a larger cross sectional study involving nearly 500 Colombian children living at two different altitudes: at 1,000 m and above 2,600 m. The main goals were to study the general development of hemoglobin and BV throughout puberty in boys and girls and the hormonal regulation of erythropoiesis by testosterone and erythropoietin. Further central points were the determination of the influences of training status and altitude on these hematological parameters as well as on VO_2_max over the entire pubertal development period and the possible dependencies between these variables. Finally, possible differences in terms of hematological and performance parameters between children and adolescents in different sports disciplines at different altitudes should be pointed out. In a first, recently published step, we showed the normal course of the increase in Hbmass during puberty in male and female adolescents and the influence of the increasing testosterone concentration in boys (Mancera-Soto et al., 2021). The work presented here represents the main part of the study and, in addition to the normal development of Hbmass, BV and VO_2_max in children and adolescents, describes the influence of training status and different altitudes on the above parameters and their interactions.

According to our first paper, Hbmass increased by 33% in girls during puberty, while an increase of 95% was observed in boys—a percentage closely associated with the occurrence of testosterone and its direct and indirect effects via lean body mass (LBM). Studies of the adaptation of Hbmass in children and adolescents through long-term training processes are difficult to perform using a longitudinal design since it is barely possible to follow a statistically sufficient number of children over several years from an untrained to a highly trained state. The few existing longitudinal studies have shown only small training effects (∼7%) over a period of 3 years [initial age 8–11 years old (Prommer et al., 2018)] and no effects over 1 year [11–15 years old (Eastwood et al., 2009)], over 18 months [15–17 years old, (Ulrich et al., 2011)] and over 3 years [12–15 years old (Landgraff and Hallén, 2020) and 16–19 years old (Steiner et al., 2019)]. In trained male adolescents, an increase in Hbmass up to the age of 21 was observed (Steiner and Wehrlin, 2011). It is undisputed that Hbmass is primarily determined by LBM, even in childhood and adolescence. However, elite adult endurance athletes also show greater Hbmass when normalized to LBM, suggesting additional, possibly training-specific influences, as is also known from the development of cardiac structures in adolescence (Bjerring et al., 2020).

Since it is hardly possible to quantify training-related effects on Hbmass and BV during all stages of puberty in a single longitudinal study with a statistically sufficient number of test subjects, we performed the present study using a cross-sectional format with a large number of endurance-trained and untrained children and adolescents in the range between 9 and 18 years old.

In addition to possible training effects, other factors influencing Hbmass must be considered. The excellent performance of endurance athletes, e.g., from Kenya and Colombia, has often been explained by their childhoods spent at moderate altitudes. While elevated Hbmass levels were not found in Kenyan runners (Prommer et al., 2010), elevated levels in Colombian cyclists have indeed been proved (Schmidt et al., 2002). Because [Hb] is much greater in higher altitude populations from the Andes than in those from East Africa (Beall et al., 2002), it seems possible that their erythropoietic system is more sensitive to hypoxia, and early training of an Andean population could have a pronounced influence on Hbmass. The first aim of this part of the project was, therefore, using a cross-sectional design with a large number of participants, to show the possible relationships of Hbmass and BV with endurance training activity and the place of residence. As in the first part of this project, a small, general effect of altitude and training on Hbmass was shown (Mancera-Soto et al., 2021); in this part, we increased the number of participants and focused on the training and altitude effects occurring selectively during pre/early puberty, mid-puberty and late puberty.

While the relationship between Hbmass level and VO_2_max has been well documented in adults, there are only a few data on such a dependency in children and adolescents. According to Wagner (Mancera-Soto et al., 2021), VO_2_max in untrained subjects is primarily limited to O_2_ consumption in muscle cells. In endurance-trained athletes, however, muscular aerobic processes are optimized, and the amount of O_2_ supplied by the blood circulation becomes increasingly a limiting factor. To date, it is not known whether Hbmass and BV might already have a performance-limiting influence in children or whether an increase in VO_2_max is based solely on muscular adaptation. The second aim of this study was therefore to investigate the extent to which a possible training-related change in Hbmass and BV could contribute to an increase in VO_2_max.

During acute stays at moderate altitude, VO_2_max decreases by ∼0.65% per 100 m (Clark et al., 2007). During stays of several weeks at moderate altitudes, the body adapts, among other ways, by increasing the Hbmass (Wachsmuth et al., 2013) but without achieving the VO_2_max at low altitudes (Schuler et al., 2007). It is known, however, that cyclists living permanently at moderate altitudes and performing on a national level achieve a similar VO_2_max at their accustomed altitudes to that of comparable athletes living at sea level or low altitudes (Schmidt et al., 2002). A third aim of this study was therefore to determine whether children and adolescents growing up at moderate altitudes might adapt to chronic hypoxia so that the altitude-related decrease in VO_2_max observed in sea-level residents can be completely compensated for.

## Materials and Methods

### Ethical Approval

Ethical approval was granted by the ethics committee of the National University of Colombia at Bogotá (reference: ID 06/2015). The study conformed to the standards set by the Declaration of Helsinki. Written informed consent was obtained from all of the children and their parents. The subjects volunteered to participate in the study and were free to withdraw at any time without any need to provide a reason.

### Subjects

In total, 475 healthy children and adolescents (girls *n* = 217, boys *n* = 258; untrained group *n* = 171, endurance trained group *n* = 304) living at two different altitudes (∼1,000 m, *n* = 204, ∼2,600 m, *n* = 271) and aged from 9 to 18 years old participated in the study. Data on Hbmass and BV in relation to serum testosterone and erythropoietin concentrations were already determined from 313 participants in a recent publication (Mancera-Soto et al., 2021). Hematological data were recorded for all 475 children, and maximum oxygen uptake (VO_2_max) was determined in 404 participants. For anthropometrical data and the exact distribution of the test subjects to the individual subgroups, see Tables 1, 2.

**TABLE 1:** Number of participants in the respective subgroups.

Sex	Training status	Altitude (m)	Pre/early puberty	Mid puberty	Late puberty
Boys	Untrained	1,000	22	8	12
		2,600	22	7	19
	Trained	1,000	10	17	46
		2,600	22	19	54
Girls	Untrained	1,000	17	9	9
		2,600	18	10	18
	Trained	1,000	20	20	14
		2,600	30	33	19

**TABLE 2:** Anthropometric data and training history.

		Pre/early puberty	Mid puberty	Late puberty	ANOVA *p* ≤ (Tanner, Sex, Interaction)
Number of boys/girls		76/85	51/72	131/60	
Age (years)	Boys	10.7 ± 1.3	13.8 ± 1.8^+++^	15.8 ± 1.5^+++^	T 0.001
	Girls	10.4 ± 1.4	13.8 ± 1.8^+++^	15.4 ± 1.7^+++^	S n.s.
					I n.s.
Body mass (kg)	Boys	36.3 ± 8.2	48.2 ± 8.4^+++^	57.3 ± 7.0^+++^	T 0.001
	Girls	34.3 ± 8.1	47.7 ± 6.9^+++^	52.2 ± 7.3^++^	S 0.001
				***	I 0.05
Height (cm)	Boys	141.9 ± 9.4	159.0 ± 8.8^+++^	168.3 ± 6.8^+++^	T 0.001
	Girls	140.1 ± 9.1	155.7 ± 6.3^+++^	159.4 ± 6.9^+^	S 0.001
			*	***	I 0.001
BMI	Boys	18.0 ± 2.5	18.9 ± 2.0^+^	20.2 ± 2.0^+++^	T 0.001
	Girls	17.3 ± 2.5	19.6 ± 2.1^+++^	20.5 ± 2.5	S n.s.
					I 0.05
Body fat (%)	Boys	16.8 ± 6.7	14.2 ± 5.3	12.8 ± 4.6	T n.s.
	Girls	18.0 ± 5.0	20.0 ± 5.6	22.0 ± 6.5	S 0.001
			***	***	I 0.001
LBM (kg	Boys	29.9 ± 5.9	41.2 ± 6.9^+++^	50.0 ± 6.0^+++^	T 0.001
	Girls	27.9 ± 5.6	38.0 ± 4.9^+++^	40.5 ± 4.7^+^	S 0.001
		*	**	***	I 0.001
Number of boys/girls		32/50	36/53	100/33	
Training volume (h week^−1^)	Boys	10.6 ± 3.9	14.1 ± 5.0^+^	16.8 ± 6.5	T 0.001
	Girls	11.8 ± 3.6	14.4 ± 5.5^+^	15.8 ± 6.9	S n.s.
					I n.s.
Training history (years)	Boys	2.4 ± 1.9	3.2 ± 2.0	4.0 ± 2.7	T 0.001
	Girls	3.0 ± 2.1	4.0 ± 2.4^+^	4.9 ± 3.0	S 0.05
					I n.s.

Values are means ±SD. The right column presents the results of the two-way ANOVA (step 1 in the statistics section). Significance of differences between boys and girls in the same stage of maturation (t-test): * = p < 0.05, ** = p < 0.01, *** = p < 0.001. Significance of differences from the previous stage of maturation (Bonferroni test): ^+^ = p < 0.05, ^+++^ = p < 0.001.

All of the trained children and adolescents practiced endurance sports, i.e., medium- and long-distance running, cycling, speed skating, race walking and triathlon. The precondition for participation in the trained group was a training history of at least 1 year, a training volume of at least 6 h per week and a training frequency of at least 3 times per week (for detailed information, see Table 2). The participants in the untrained group did not practice any other sport apart from school sports. The subjects were recruited in the Colombian regions around Bogotá (Central East Andean region, ∼2,600 m above sea level) and Tuluá/Cali (West Andean region, ∼1,000 m). Ethnicity of the participants corresponded to the regional distribution, which differed only slightly between both regions [Central East/West Andes: African origin 5.1%/14.0%, European origin 58.9%/55.4%, Native American origin: 36.0%/30.5% (Ossa et al., 2016)]. With the exception of 17 participants from the Central-East group who had lived in Bogotá for at least 5 years, they had lived their entire lives at their respective altitudes and had not changed their altitude for more than a week over the past year.

None of the female participants indicated the use of hormonal contraceptives, and none used iron, folic acid or any supplements that could affect the parameters determined in this study.

### Study Design

This cross sectional study with an independent groups design was conducted in Bogotá and in Tuluá on two consecutive days. On the first day, a medical examination was performed, followed by an anthropometric evaluation. Hemoglobin mass was determined using the CO-rebreathing method according to Schmidt and Prommer, (2005) and Prommer and Schmidt, (2007) and a cubital venous blood sample; i.e., 4 ml of heparinized blood were obtained for the determination of basic hematological parameters. On the second day, an incremental step test on a cycle ergometer or treadmill was performed to assess VO_2_max. The cyclists, speed skaters, race walkers and triathletes, as well as the untrained children, were tested on a cycle ergometer, and the runners were tested on a treadmill.

### Medical Examination and Anthropometrical Evaluation

Before the study, the children and their parents were advised not to practice exhaustive sports before both test days, to sleep at least 8 h, and to eat carbohydrate-rich meals. Health status was checked by medical examination, including electrocardiography, under resting conditions. The stage of biological maturation was evaluated using the method of coevaluation by a physician and self-report, according to Tanner, (1962), and the children were classified in stages I to V according to their external primary and secondary sexual characteristics.

For the anthropometrical evaluation, skinfold caliper measurements were performed by an identical scientist using the triceps, subscapular, supra-iliac, abdominal, thigh and medial calf (Stewart and Marfell-Jones, 2011). Percentage body fat and absolute lean body mass were estimated using a correction for the age and gender of the participants (Slaughter et al., 1988).

### Sample Transport and Storage

The whole procedure, including blood sampling, sample transport, sample storage and sample analyses, was performed under standardized conditions oriented to current WADA guidelines (WADA, 2019). Four milliliters of blood were drawn from a cubital vein using EDTA vacutainers after the subject was left in a sitting position for at least 15 min. The samples were stored in a +4°C refrigerator until they were transported within 24 h under controlled, cool conditions to the WADA-related Institute for Doping Analysis in Bogotá, Colombia.

### Blood Analytical Procedures

The hematological measurements were obtained at the Institute of Doping Analysis in Bogotá. A Sysmex XT 2000i hematological analyzer (Sysmex, Norderstedt, Germany) was used for the determination of hemoglobin concentrations ([Hb]) and hematocrit (Hct).

### Hemoglobin Mass Determination

Hemoglobin mass (Hbmass) was determined using the CO-rebreathing method as described by Schmidt and Prommer, (2005) and modified by Prommer and Schmidt, (2007). Briefly, a bolus of 99.97% carbon monoxide (boys and girls < 14 years old—untrained 0.7 ml/kg body mass, trained 0.8 ml/kg; boys > 14 years old—untrained 1.0 ml/kg, trained 1.2 ml/kg; girls > 14 years old—untrained 0.8 ml/kg, trained 1.0 ml/kg) was administered to subjects and rebreathed along with 2–3 L of 100% O_2_ for 2 min. The COHb concentration was measured in sextuplicate before and 7 min after starting the CO-rebreathing in capillarized blood from an earlobe using the CO-oximeter OSM3 (Radiometer, Copenhagen, Denmark). The total Hbmass was then calculated from the difference in the COHb concentration before and after the rebreathing maneuver. A more detailed description was previously provided by Mancera-Soto et al., (2021). During the whole study, identical equipment was used by the same staff. The typical error (TE) of this method obtained in our laboratory was 2.2% and was in accordance with the TE published by Gore et al., (2005). BV, red cell volume (RCV) and plasma volume (PV) were calculated using Hb mass, venous [Hb] and venous hematocrit values (obtained from the Sysmex XT 2000i system) as described by Schmidt and Prommer, (2005).

### VO_2_max Tests

VO_2_max was determined by an incremental step test until subjective exhaustion. Except for the runners, all of the trained and untrained children were tested on the identical cycle ergometer (Monarc Ergomedic 839, Monark Exercise AB, Vansbro, Sweden). The reason why the untrained children were tested on the bicycle ergometer was that they could be graded much more precisely than on the treadmill. Among the trained children, cyclists, triathletes and speed skaters were sufficiently familiar with bicycles to reach their discipline-specific maxima, while runners were tested on a treadmill (HP Cosmos Quasar, h/p/cosmos sports and medical GmbH, Nussdorf—Traunstein, Germany) to obtain their discipline-specific maximum values. Since in untrained subjects and in moderately trained endurance athletes, the VO_2_max on the treadmill is 5–10% higher than on the bicycle ergometer because of the greater muscle mass involved (Millet et al., 2009), the values of the runners were corrected by 7% (Hermansen and Saltin, 1969). Before starting the testing, all of the children were familiarized with the ergometer or treadmill. In all of the tests, a 3-min warm-up was provided with the initial setting, and the load was increased differently in the individual groups according to the scheme presented in the supplemental material. The test was ended when the participant could not maintain the number of revolutions (cycle ergometer) or speed (treadmill). Ninety-seven percent reached at least two of the following criteria: 1) occurrence of a plateau of VO_2_ despite an increase in exercise intensity; 2) heart rate greater than 180 beats/min; and 3) respiratory exchange ratio (RER) > 1.1. Measurement of VO_2_max was always performed using the identical portable spirometry system (COSMED Model Quark CPET, COSMED, Rome, Italy). For VO_2_max, values were averaged over a period of 30 s before exhaustion.

### Statistics

For statistical analysis, IBM SPSS software, version 28, was used. Data refer to the stage of biological maturation and are presented as the means and standard deviations for the respective stage of puberty. To increase the statistical power, we pooled the data on Tanner stages I (pre puberty) and II (early puberty) and named them pre/early puberty, as well as stages IV and V (late puberty), and compared them with Tanner stage III (mid puberty). These pooled data are presented in this paper, and the data divided into the five Tanner subgroups are shown in the supplemental material.

The following methods were used for the statistical analysis.

1) In the first step, changes in the hematological variables and VO_2_max over the course of pubertal development were examined. For the hematological parameters, a two-way ANOVA with puberty and sex as the independent variables was used. For VO_2_max, training status was added as an additional independent variable, so a three-way ANOVA was used here.
2) Additionally, a one-way ANOVA with puberty as a single independent factor was performed to show the development during puberty separately in boys and girls, as well as for VO_2_max separately in the trained and untrained groups. The significance of mean differences was tested using the Bonferroni test when comparing more than two groups (comparison of the three pubertal stages) and using the unpaired t-test when comparing two groups (e.g., sex differences or differences between the trained and untrained groups within one pubertal stage). To exclude the possible influences of different group sizes, effect sizes (Cohen’s d) were calculated with pooled standard deviations to judge the differences between the individual puberty, sex and training groups (Sullivan and Feinn, 2012), where d = 0.2 presents small, d = 0.5 presents medium, and d = 0.8 presents large effects (Lakens, 2013).
3) Multifactorial ANOVA followed by multiple regression analyses were conducted with the whole group to determine and quantify the possible influences of puberty, sex, altitude and training status on the dependent variables of Hbmass, BV and PV. The same procedure was performed to determine the effects of puberty, sex, altitude, training status, and Hbmass on VO_2_max.
4) The same procedure used for the whole group was applied separately to the pre/early-, mid-, and late puberty groups, and the main effects were calculated for each independent variable.
5) Bivariate linear regression analyses were performed with Hbmass as the dependent variable and LBM as the independent variable and with VO_2_max as the dependent variable and Hbmass as the independent variable.

## Results

The anthropometric characteristics show the well-known time course during development, with higher fat mass in girls than in boys in the mid and late puberty and a larger LBM in the boys than in the girls during these stages of maturation (Table 2).

### Hemoglobin and Blood Volumes

In both sexes, Hbmass was elevated in the later puberty stages compared to pre/early puberty; this was, however, much more pronounced in the boys (from 408 ± 88 g to 788 ± 133 g) than in the girls (from 367 ± 86 g to 531 ± 107 g; Table 3). Significantly higher absolute Hbmass and blood volumes (RCV, BV, PV) were observed in girls during mid puberty compared to pre/early puberty, and in boys during late puberty compared to pre/early and mid puberty. When Hbmass, RCV, BV and PV were normalized to LBM, no difference or only small differences were detected between girls and boys at pre/early puberty, and no significant difference existed between the female puberty groups. In boys, however, considerably higher values for Hbmass, RCV and BV were detected in the mid puberty and late puberty groups compared to pre/early puberty (Table 3). The PV did not differ between the sex and puberty groups with the exception of higher values in mid-puberty boys (Table 3).

**TABLE 3:** Hematological data and blood volumes in boys and girls in different stages of sexual maturation.

		Pre/early puberty	Mid puberty	Late puberty	ANOVA *p* ≤ (Tanner, Sex, Interaction)
number of boys/girls		76/85	51/72	131/60	
[Hb] (g dl^−1^)	Boys	14.6 ± 1.0	15.1 ± 1.2^+++^; *0.5*	15.8 ± 1.2^+++^; 0.6	T 0.001
	Girls	14.3 ± 0.9	14.2 ± 1.0; 0.1	14.2 ± 1.1; 0.0	S 0.001
	d_cohen_	0.3	***; 0.8	***; 1.5	I 0.001
Hct (%)	Boys	42.0 ± 2.3	43.9 ± 2.9^+++^; 0.7	46.5 ± 3.0^+++^; 0.9	T 0.001
	Girls	41.8 ± 2.3	42.2 ± 2.6; 0.2	42.3 ± 3.1; 0.0	S 0.001
	d_cohen_	0.1	***; 0.6	***; 1.4	I 0.001
Hbmass (g)	Boys	408 ± 88	636 ± 152^+++^; 1.9	788 ± 133^+++^; 1.1	T 0.001
	Girls	367 ± 86	503 ± 104^+++^; 1.4	531 ± 107; 0.3	S 0.001
	d_cohen_	**; 0.5	***; 1.1	***; 2.2	I 0.001
Hbmass (g kg^−1^)	Boys	11.4 ± 1.5	13.1 ± 1.8^+++^; 1.0	13.8 ± 1.5^+^; 0.4	T 0.001
	Girls	10.8 ± 1.4	10.6 ± 1.7; 0.1	10.2 ± 1.3; 0.3	S 0.001
	d_cohen_	*; 0.4	***; 1.5	***; 2.5	I 0.001
Hbmass (g kgLBM^−1^)	Boys	13.7 ± 1.5	15.3 ± 1.7^+++^; 1.0	15.8 ± 1.4; 0.3	T 0.001
	Girls	13.2 ± 1.6	13.2 ± 1.9; 0.0	13.0 ± 1.5; 0.1	S 0.001
	d_cohen_	*, 0.3	***; 1.5	***; 2.0	I 0.001
RCV (ml)	Boys	1,182 ± 260	1849 ± 452^+++^; 1.9	2,320 ± 399^+++^; 1.1	T 0.001
	Girls	1,082 ± 259	1,490 ± 307^+++^; 1.4	1,581 ± 326; 0.3	S 0.001
	d_cohen_	*; 0.4	***; 1.0	***; 2.0	I 0.001
RCV (ml kg^−1^)	Boys	32.7 ± 4.6	38.2 ± 5.2^+++^; 1.1	40.5 ± 4.5^+^; 0.5	T 0.001
	Girls	31.7 ± 4.1	31.3 ± 5.0; 0.1	30.3 ± 4.3; 0.2	S 0.001
	d_cohen_	0.2	***; 1.4	***; 2.3	I 0.001
RCV (ml kgLBM^−1^)	Boys	39.4 ± 4.6	44.5 ± 5.1^+++^; 1.1	46.3 ± 4.2; 0.4	T 0.001
	Girls	38.7 ± 4.8	39.1 ± 5.6; 0.1	38.9 ± 4.8; 0.0	S 0.001
	d_cohen_	0.1	***; 1.0	***; 1.7	I 0.001
BV (ml)	Boys	3,092 ± 661	4,622 ± 1050^+++^; 1.8	5,495 ± 931^+++^; 0.9	T 0.001
	Girls	2,836 ± 657	3,883 ± 756^+++^; 1.5	4,112 ± 770; 0.3	S 0.001
	d_cohen_	*; 0.4	***; 0,8	***; 1.6	I 0.001
BV (ml kg^−1^)	Boys	85.4 ± 10.4	95.6 ± 11.4^+++^; 0.9	95.9 ± 10.1; 0.0	T 0.01
	Girls	83.5 ± 11.5	81.6 ± 11.9; 0.2	78.8 ± 10.2; 0.3	S 0.001
	d_cohen_	0.2	***; 1.2	***; 1.7	I 0.001
BV (ml kgLBM^−1^)	Boys	102.9 ± 10.8	111.6 ± 12.4^+++^; 0.8	109.6 ± 9.4; 0.2	T 0.01
	Girls	101.9 ± 13.0	102.0 ± 13.3; 0.2	101.3 ± 12.2; 0.1	S 0.001
	d_cohen_	0.1	***; 0.7	***; 0.8	I 0.01
PV (ml)	Boys	1909 ± 414	2,772 ± 629^+++^; 1.7	3,175 ± 581^+++^; 0.7	T 0.001
	Girls	1756 ± 409	2,393 ± 473^+++^; 1.4	2,531 ± 479; 0.3	S 0.001
	d_cohen_	*; 0.4	***; 0.7	***; 1.42	I 0.001
PV (ml kg^−1^)	Boys	52.7 ± 6.5	57.4 ± 7.3^+++^; 0.7	55.4 ± 7.0; 0.3	T n.s.
	Girls	51.8 ± 7.9	50.3 ± 7.5; 0.2	48.6 ± 6.9; 0.2	S 0.001
	d_cohen_	0.1	***; 1.0	***; 1.0	I 0.001
PV (ml kgLBM^−1^)	Boys	63.6 ± 7.0	67.1 ± 8.7^+^; 0.5	63.4 ± 7.0^+^; 0.5	T n.s.
	Girls	63.2 ± 8.9	62.8 ± 8.6; 0.0	62.4 ± 8.6; 0.0	S 0.05
	d_cohen_	0.1	**; 0.5	0.1	I n.s.

The data are presented as absolute values and as values normalized to body mass and lean body mass (LBM). Hbmass, hemoglobin mass; RCV, red cell volume; BV, blood volume; PV, plasma volume. The right column presents the results of the two-way ANOVA (step 1 in the statistics section). Significance of differences between boys and girls in the same stage of maturation (t-test): *p < 0.05, **p < 0.01, ***p < 0.001, Significance of differences from the previous stage of maturation (Bonferroni test): +*p* < 0.05, ++*p* < 0.01, +++*p* < 0.001. The effect size for the comparison between boys and girls, as well as for the comparison of different stages of maturation, is presented in italics as d_cohen_ next to the symbols for significance.

Hbmass, hemoglobin mass; RCV, red cell volume; BV.blood volume; PV, plasma volume.

Hemoglobin concentrations ([Hb]) in the boys was higher at late puberty (15.8 ± 1.2 g ^.^ dl^−1^) than at pre/early puberty (14.6 ± 1.0 g ^.^ dl^−1^) while in the girls, no difference was observed among the different puberty groups (Table 3). At pre/early puberty, no sex-related differences in [Hb] and Hct were detected, while at mid puberty and late puberty, all of the hematological values were considerably higher in the boys (Table 3).

Multifactorial ANOVA (see #3 in the section “statistics”) with absolute Hbmass and with normalized Hbmass (g kg^−1^ and g kgLBM^−1^) as the dependent variables yielded significant main effects of phase of puberty, sex, training status and place of residence, as well as of the interaction of phase of puberty with sex and training status (Table 4). No interaction was found for the phase of puberty with place of residence.

**TABLE 4:** Multiple analyses of variance with hematological parameters **(A)** and VO_2_max **(B)** as dependent variables followed by a multiple regression analysis.

A	Whole group *n* = 475					
	Main effect				Interaction between	
	Puberty Pre-, mid-, end-puberty	Sex Boys/girls	Training Trained/untrained	Altitude 2600m/1000 m	Sex and puberty	Training and puberty
[Hb] (g dl^−1^)	0.3 ± 0.1***	2.3 ± 0.1***	n.s.	1.0 ± 0.1***	***	
Hct (%)	1.3 ± 0.1***	2.3 ± 0.2***	0.8 ± 0.2**	2.3 ± 0.2***	***	
Hbmass (g)	138 ± 7***	143 ± 11^***^	65 ± 11***	35 ± 11***	***	*
Hbmass (g kg^−1^)	0.41 ± 0.1***	2.2 ± 0.1***	1.4 ± 0.1***	0.7 ± 0.1***	***	*
Hbmass (g kgLBM^−1^)	0.44 ± 0.1***	1.8 ± 0.1***	1.0 ± 0.1***	0.8 ± 0.1***	***	*
BV (ml)	924 ± 46***	764 ± 78***	438 ± 81***	n.s.	***	*
BV (ml kg^−1^)	n.s.	10.6 ± 1.0***	9.3 ± 1.0***	n.s.	***	*
BV (ml kgLBM^−1^)	n.s.	5.7 ± 1.1***	6.5 ± 1.1***	n.s.	***	*
PV (ml)	508 ± 28***	365 ± 48***	231 ± 49***	-95 ±47*	***	
PV (ml kg^−1^)	n.s.	4.6 ± 0.6***	5.0 ± 0.7***	-2.1 ± 0.6**		
PV (ml kgLBM^−1^)	n.s.	n.s.	3.2 ± 0.8***	-2.7 ± 0.7***		

B	Whole group n = 404						
	Puberty Pre-, mid-, end-puberty	Sex Boys/girls	Training Trained/untrained	Altitude 2600m/1000 m	Hbmass g or g/kg or g/kgLBM	Interaction between training and Hbmass
VO_2_max (ml min^−1^)	91 ± 25***	201 ± 36***	531 ± 33***	n.s.	2.8 ± 0.1***	***
VO_2_max (ml min^−1^ kg^−1^)	-1.2 ± 0.4**	5.1 ± 0.8***	12.4 ± 0.8***	1.7 ± 0.7***	2.0 ± 0.2***	*
VO_2_max (ml ^**.**^ min^−1^ kgLBM^−1^)	-1.2 ± 0.5**	5.9 ± 0.9***	14.5 ± 0.8***	2.4 ± 0.8***	1.2 ± 0.2***	**

Presented are significant main effects with standard errors and interactions. The effects were calculated as described in step 3 in the statistics section by multiple regression analysis. [Hb], hemoglobin concentration; Hct, hematocrit; Hbmass, hemoglobin mass; BV, blood volume; PV, plasma volume; LBM, lean body mass. Significance of effects: *p < 0.05, **p < 0.01, ***p < 0.001, n.s., not significant.

[Hb], hemoglobin concentration; Hct, hematocrit; Hbmass, hemoglobin mass; BV, blood volume; PV, plasma volume; LBM, lean body mass; n.s., not significant.

**FIGURE 1:**
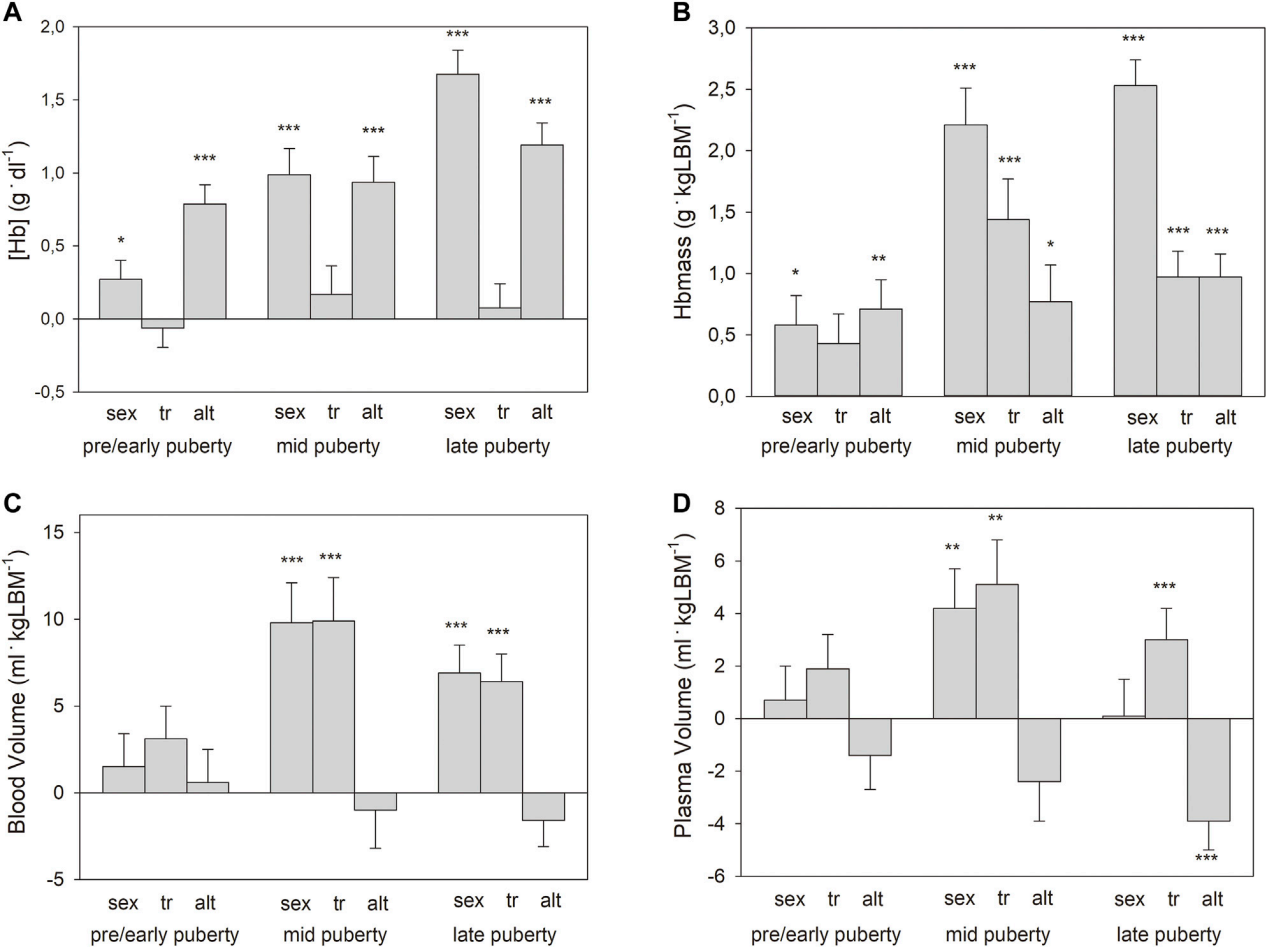
Main effects with standard errors calculated by multiple regression analysis separately for the pooled puberty stages for sex (boys vs. girls), training status (tr, trained vs. untrained), and altitude (alt, 2,600 m vs. 1,000 m) on: **(A)** hemoglobin concentrations ([Hb]); **(B)**. Hbmass; **(C)**. blood volume; and **(D)**. plasma volume. Data for Hbmass, BV, and PV are normalized to lean body mass (LBM). Significance of effects: * = *p* < 0.05, ** = *p* < 0.01, *** = *p* < 0.001.

When the whole group was divided and the pre/early-, mid- and late pubertal phases were separately analyzed (see #4 in the section “statistics”), only a slight, significant effect of sex and no effect of training status were observed in the pre/early pubertal group for absolute and normalized Hbmass, while both effects were highly significant in the later stages. (Figure 1B shows Hbmass normalized to kg LBM). Moderate altitudes had significant, positive effects in all puberty stages (Figure 1B).

Multifactorial ANOVA (see #3 in the section “statistics”) for BV as a dependent variable showed significant, positive main effects of sex and training status, as well as interactions for sex and training status, with the phase of puberty but no effect of altitude (Table 4). The main effects of sex and training status (see #4 in the section “statistics”) were highly pronounced in the mid- and late pubertal stages but did not exist pre/early puberty (Figure 1C). The effects of sex and training status on PV were similar to those on BV, while moderate altitude exerted a negative effect on PV (Table 4; Figure 1D).

### Maximum Oxygen Uptake (VO_2_max)

The absolute VO_2_max showed a well-known sex- and training-specific increase with increasing sexual maturity (Table 5). When VO_2_max was normalized to LBM, there was still a clear, positive influence of sex and training status but not of the puberty stage.

**TABLE 5:** VO_2_max in trained and untrained boys and girls in different stages of sexual maturation.

			Pre/early puberty	Mid puberty	Late puberty	ANOVA *p* ≤ (Tanner, Sex, Training, Interaction)
Number of boys/girls		Untrained	43/32	13/17	28/19	
		Trained	29/40	32/37	87/27	
VO_2_ max (ml ^**.**^ min^−1^)	Untrained	Boys	1,561 ±427	2,141 ± 395^+++^; *1.4*	2,408 ± 447; *0.6*	T 0.001
		Girls	1,365 ±355	1,624 ± 350^+^; *0.7*	1,704 ± 299; *0.2*	S 0.001
		d_cohen_	*; 0.6	***; 1.4	***; 1.8	Tr 0.001
	Trained	Boys	1,981 ± 483	2,900 ± 521^+++^; *1.8*	3,449 ± 463^+++^; *1.1*	T × S 0.001
		Girls	1,660 ±328	2,296 ± 290^+++^; *2.0*	2,337 ± 383; *0.1*	T × Tr 0.001
		d_cohen_	**; 0.8	***; 1.5	***; 2.5	S × Tr 0.05
VO_2_ max (ml min^−1^ kg^−1^)	Untrained	Boys	43.7 ± 11.2	42.9 ± 4.7; *0.1*	42.3 ± 6.4; *0.1*	T n.s.
		Girls	39.5 ± 6.5	33.7 ± 5.6^++^; *0.9*	31.8 ± 3.7; *0.4*	S 0.001
		d_cohen_	*; 0.4	***; 1.8	***; 1.9	Tr 0.001
	Trained	Boys	55.8 ± 7.2	61.0 ± 8.1^++^; *0.7*	60.5 ± 6.2; *0.1*	T × S 0.001
		Girls	49.6 ± 8.4	48.9 ± 5.1; *0.1*	46.9 ± 5.9; *0.4*	T × Tr 0.001
		d_cohen_	**; 0.8	***; 1.8	***; 2.2	S × Tr n.s.
VO_2_ max (ml ^**.**^ min^−1^^**.**^ kgLBM^−1^)	Untrained	Boys	53.3 ± 11.8	52.5 ± 7.2; *0.1*	49.8 ± 6.7; *0.4*	T n.s.
		Girls	48.9 ± 7.9	43.4 ± 7.1^+^; *0.7*	42.5 ± 3.8; *0.2*	S 0.001
		d_cohen_	*0.4*	**; 1.9	***; 1.3	Tr 0.001
	Trained	Boys	66.0 ± 8.7	69.9 ± 7.7; *0.5*	68.8 ± 6.3; *0.2*	T × S 0.05
		Girls	59.9 ± 8.7	60.2 ± 5.8; *0.0*	58.1 ± 6.8; *0.3*	T × Tr 0.01
		d_cohen_	**; 0.7	***; 1.4	***; 1.7	S × Tr n.s.

The data are presented as absolute values and as values normalized to body mass and lean body mass (LBM). Significance of differences between boys and girls in the same stage of maturation: * = p < 0.05, ** = p < 0.01, *** = p < 0.001. The right column presents the results of the two-way ANOVA (step 1 in the statistics section). Significance of difference from the previous stage of maturation (Bonferroni test): + = p < 0.05, ++ = p < 0.01, +++ = p < 0.001. Significance of differences between trained and untrained groups in the same stage of maturation (t-test): p < 0.001 in all cases. The effect size for the comparison between boys and girls, as well as for the comparison of different stages of maturation, is presented in italics as dcohen next to the symbols for significance. The effect size for the comparison between the trained and untrained groups in all cases: dcohen > 0.8.

**FIGURE 2:**
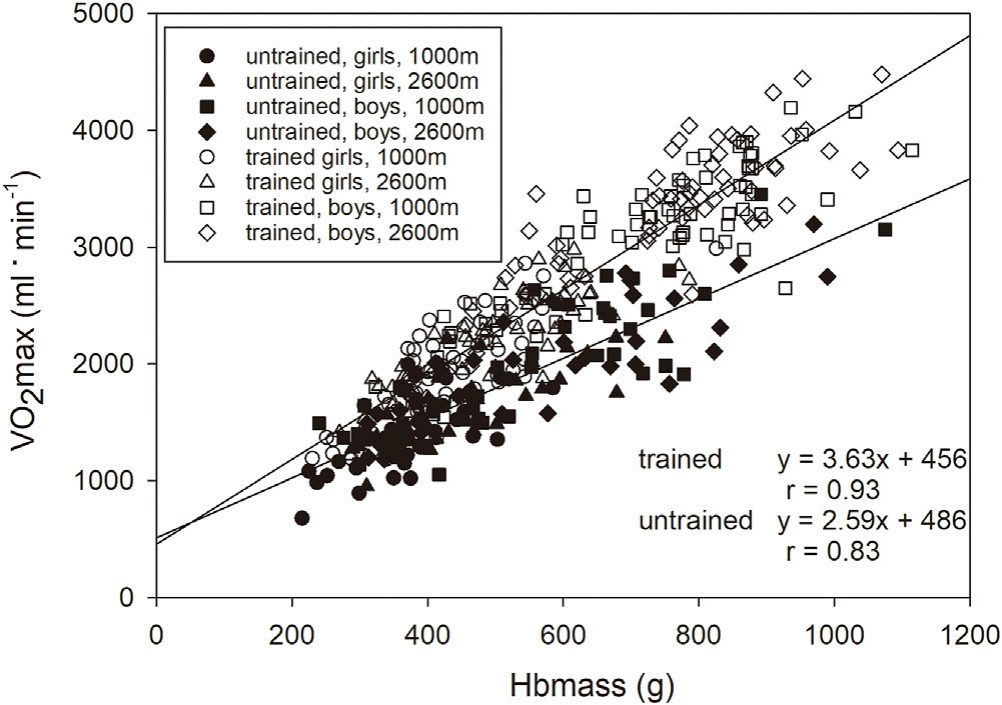
Relationship between Hbmass and VO_2_max normalized to LBM presented for the trained (y = 2.3x + 31.0, r = 0.54, *p* < 0.001) and untrained groups (y = 1.4x + 30.4, r = 0.31, *p* < 0.01).

The linear regression analysis between Hbmass and VO_2_max showed a clear difference between the trained and untrained groups, with a steeper slope of the regression line of the trained group (3.63 vs. 2.59. The relationship between the two variables became weaker when the values related to LBM were used (trained, absolute vs. normalized: r = 0.93 vs r = 0.54, untrained: r = 0.83 vs r = 0.30), but the difference between the groups persisted (Figure 2).

Multifactorial ANOVA (see #3 in the section “statistics”) with the absolute VO_2_max of the entire group as the dependent variable showed significant main effects for puberty status, sex, training status, and Hbmass and interactions between training status and Hbmass (Table 4). For normalized VO_2_max as the dependent variable, also a small positive effect was found for altitude.

**FIGURE 3:**
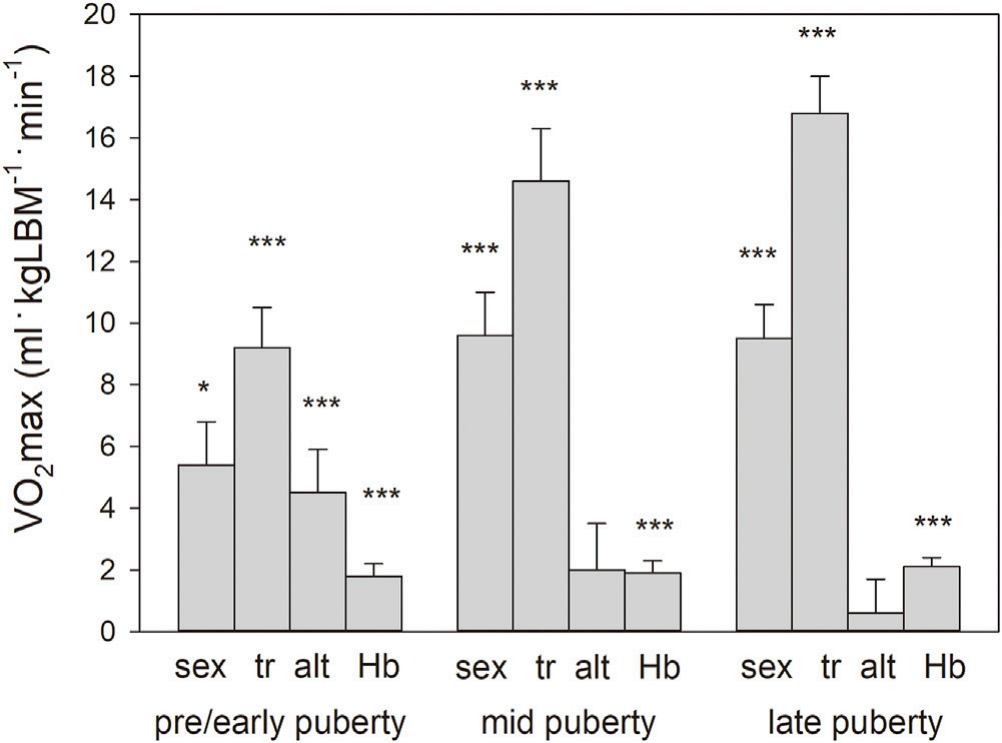
Main effects with standard errors calculated by multiple regression analysis separately for the pooled puberty stages for sex (boys vs girls), training status (tr, trained vs. untrained), altitude (alt, 2,600 m vs. 1,000 m), and Hbmass (g kgLBM^−1^) on VO_2_max normalized to lean body mass. Because of the interaction in the mid-and late puberty groups, the main effects for sex and Hbmass were calculated independently. Significance of effects: * = *p* < 0.05, *** = *p* < 0.001.

In all stages of puberty, there were significant main effects (see #4 in the section “statistics”) of the confounding factors of sex, training, and Hbmass. Moderate altitude also tended to have a positive effect on VO_2_max. For the magnitude of the main effects, see Figure 3.

## Discussion

In our recent study conducted with ∼65% of the participants of this study, we monitored the normal development of Hbmass and BV during childhood and adolescence and the role that testosterone plays during male puberty. In this part of the project, we demonstrated that an association of Hbmass and BV with training status does not exist until the onset of puberty, but close associations are found thereafter. Living at a considerable altitude, in contrast, stimulates Hbmass age independently.

An important result is that the training status determines VO_2_max much more via other factors than via Hbmass and that VO_2_max is influenced by an endurance training-associated increase in Hbmass only after the onset of puberty. Living at a moderate altitude completely compensates for the hypoxic conditions and does not negatively affect VO_2_max.

### Hemoglobin and Blood Volumes During Sexual Maturation

The development of Hbmass, BV and PV during maturation corresponds to the data we recently published (Mancera-Soto et al., 2021). In girls, there was an equal percentage increase in RCV or Hbmass and PV by approximately 45% for both parameters from pre/early to late puberty, reflected in unchanged [Hb] and Hct levels. However, in boys, Hbmass increased by 95%, while PV increased by only 65%, resulting in an increase in [Hb] from 14.6 g dl^−1^–15.8 g dl^−1^. These data clearly show that [Hb] is not a good indicator of the absolute amount of hemoglobin in the body and does not represent the quantitative development of hemoglobin in childhood and adolescence.

### Hbmass and Training

The influence of training has only been investigated in a few studies with a small number of test subjects. Prommer et al., (2018) described a small training effect of 25 g (7%) over the course of 2.5 years in children aged 8–11 years old at the start of the study, whereas other studies did not find any specific training effects in 12–15-year-old boys and girls (Landgraff and Hallén, 2020) or in 15–17-year-old boys (Ulrich et al., 2011).

**FIGURE 4:**
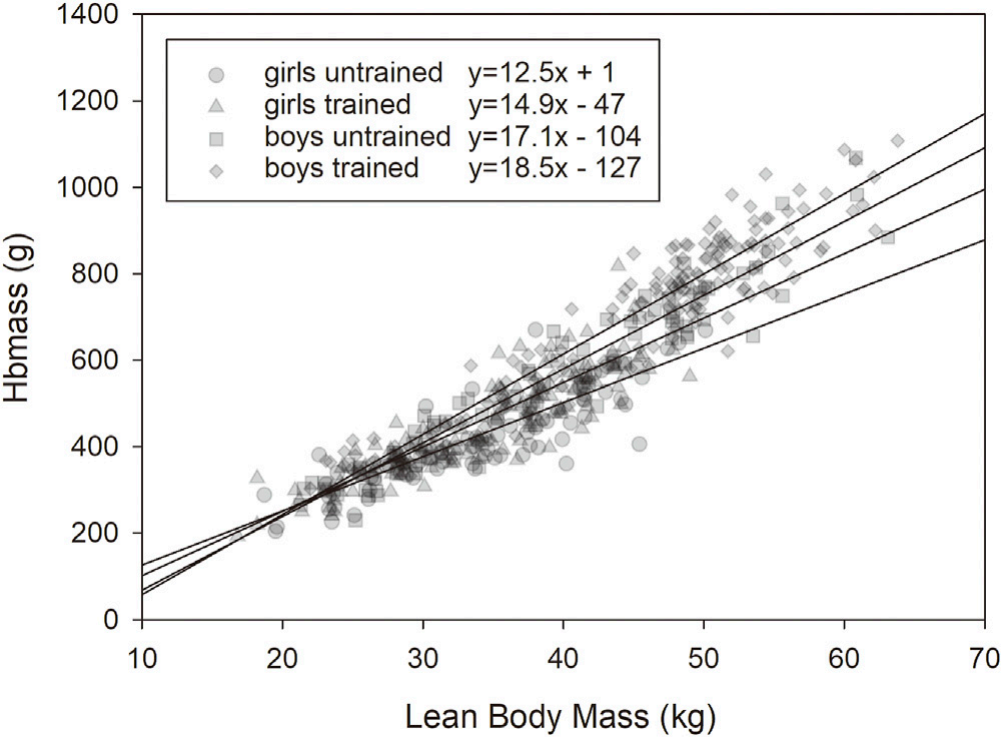
Relationship between lean body mass (LBM) and Hbmass in the trained and untrained male and female groups.

As demonstrated in Figure 1, at pre/early puberty, there were no associations between training status and Hbmass or blood volumes. In the mid- and late pubertal states, however, there were significant effects ranging between 10 and 12% occurring independently on LBM in both sexes (Figures 1, 4). Possible reasons for these delayed training adaptations could be the training status of the different age groups, although the youngest participants already had a training history of at least 1 year (mean 2.7 years) and a training volume of at least 6 h/week (mean 11 h/week) completed. In contrast, the mean training history and the weekly training volume in the end-pubertal group were up to twice as high (see Table 2).

Conversely, the relatively small increase in Hbmass at later developmental ages, which is only slightly greater than in adults who begin intensive endurance training (Schmidt and Prommer, 2008), demonstrates only slight erythropoietic stimulation from exercise. Since there was no interaction between training status and sex, the increase in Hbmass in the mid- and late pubertal training groups was not achieved through training-related testosterone stimulation. This outcome is in line with our recently published data, which showed no association between training status and serum testosterone levels during puberty (Wagner, 2000).

BV shows a very similar association with training status as Hbmass, which could indicate that the increase in Hbmass occurs less via primary erythropoietic stimulation but rather as a consequence of the training-related increase in BV with subsequent normalization of [Hb] and Hct *via* a so-called critmeter (Montero and Lundby, 2018). This outcome would mean that the undoubtedly higher and pronounced Hbmass values in elite endurance athletes are mainly genetically determined and can only be modulated to a relatively small extent by training. However, data on the genetic mechanisms are scarce. Studies with endurance athletes have identified only NFIA-AS2 polymorphisms as a mediator of erythropoiesis, with carriers of the variant genotype having higher hemoglobin mass, BV, and PV (Malczewska-Lenczowska et al., 2020; Kim et al., 2022). The view of the involvement of the kidney as a critmeter is also supported by the behavior of the PV, which was significantly increased in the mid- and late puberty training groups and thus, together with the larger erythrocyte volume, contributes to the increase in BV.

### Hbmass and Altitude

The influence of moderate altitude on the [Hb] is approximately 1.0 g/dl in all stages of puberty and does not show sex-specific characteristics, corresponding to data from Böning et al., (2004); Schmidt et al., (2002) in adults from the same regions, as well as data from Gassmann et al., (2019), who showed significantly greater [Hb] in inhabitants of the Andes compared to inhabitants of similar high altitudes from Tibet and East Africa. The influence of altitude on Hbmass and BV has not yet been investigated in children. For the first time, we showed here that an equal erythropoietic adaptation as in adults also occurs in childhood and adolescence. There are data available from adults from the same regions where this study was conducted showing almost the same absolute Hbmass values and altitude-related differences in women as we do here in Bogotá for late pubertal girls (Böning et al., 2004). In men, however, trained and untrained subjects from Bogotá show significantly higher values than adolescents during late puberty (Schmidt et al., 2002). This outcome demonstrates that, in contrast to the girls, the development of Hbmass and the associated BV is not yet complete at the end of puberty in boys, which is in good agreement with the data of Steiner and Wehrlin, (2011), who observed an increase in Hbmass up to the age of 21 years old.

In our recently published study, we did not find any altitude-related effects on serum erythropoietin and testosterone concentrations in children and adolescents (Wagner, 2000) and can therefore exclude a simple increase in these hormones as the cause of the higher Hbmass at higher altitudes. However, it seems possible that, at higher altitudes, there occurs a decrease in the soluble EPO receptor, improving the binding of EPO to its membrane receptor (Villafuerte et al., 2014) and thereby augmenting the erythropoietic effectiveness at identical serum EPO concentrations.

The whole BV did not differ between the groups from low and moderate altitudes since the higher erythrocyte volume at moderate altitudes is compensated for by a lower PV (Table 3; Figure 1), which can be attributed to a hypoxia-related change in the concentration of the volume-regulating hormones aldosterone and ANP (Schmidt et al., 1999). In adults, this behavior of the PV has not only been described for short-term stays at different altitudes (Beidleman et al., 2017) but also for chronic stays at the identical moderate altitudes to those in this study (Böning et al., 2004).

In this study, we did not detect any interactions between the training effects and the altitude effects on Hbmass but only the main effects, which are demonstrated in Figure 1. This outcome suggests independent regulatory mechanisms, which at moderate altitudes are likely to be hypoxic renal stimulation and, with regard to the training effects, compensation for the expansion of the PV (Schmidt et al., 1988).

### VO_2_max and Training

The increase in the absolute VO_2_max with age, as well as the sex differences, correspond to the model developed by Armstrong and Welsman, (2019). Since LBM is the main influencing factor on VO_2_max (Armstrong and Welsman, 2019), the normalized VO_2_max should be constant during the maturation and growth process of children and adolescents. Landgraff et al., (2021), however, demonstrated a slight decrease over the course from 12 to 15 years of age in endurance-trained boys. Our data do not show any significant differences in the normalized values during the growth and maturation process but a clear trend toward decreasing values with increasing age in the untrained children and increasing values in the trained children. Because in our study, the normalized data differed greatly between the trained and untrained children, factors other than LBM also influenced VO_2_max and even made variation in the normalized values during maturation likely.

The improvements through training shown by Bar-Or for children and adolescents (Bar-Or and Rowland, 2004) were less than 5% in pre/early pubescent children and more than 15% in mid- and late pubertal adolescents. This different behavior in the age groups could in principle also be observed in this study. However, the differences between the trained and untrained groups in all stages of development were greater than those in Bar-Or’s review and amount to ∼20% in the pre/early pubertal stage and ∼40% in the mid- and late pubertal stages for both sexes (Table 5). While Bar-Or does not provide any definitive causes for better trainability at older ages, the finding in our study could also be due to a longer training history and possibly more intensive training in the older children.

To date, VO_2_max has been examined several times in children and adolescents, but the importance of both Hbmass and BV on VO_2_max has rarely been quantified. In statistical analysis, Hbmass and BV have almost the same effect on VO_2_max. Both parameters are associated with the maximum stroke volume (Schierbauer et al., 2021), and a high Hbmass guarantees an advantageous [Hb] and thus O_2_ transport capacity. In children, both maximum SV and $a-\bar{v}\;O_{2}\;\text{diff}$ are mainly determined by the magnitude of LBM (Armstrong and Welsman, 2020). The effects of endurance training on VO_2_max, in contrast, are—like in adults (Lundby et al., 2017)—mainly brought about by increasing the SV and less so by increasing the $a-\bar{v}\;O_{2}\;\text{diff}$ (Obert et al., 2003). As a reason for this change, cardiac remodeling through the development of cardiac morphology and function in youth athletes has been reported (Nottin et al., 2002). Recently, Bjerring et al., (2020) demonstrated distinct phases in the development of the young athletic heart with a tendency toward concentric remodeling in endurance athletes at the age of 12, with increased wall thickness and cardiac mass compared to sedentary peers. In later ages, this development changed to eccentric remodeling. Cardiac changes during adolescence appear to be associated with the development of BV and Hbmass, as Perkins et al., (2022) showed a simultaneous increase in left ventricular mass and expansion of Hbmass and BV in trained children in the later stages of puberty.

In this study, the effect of the training status on VO_2_max, already present in the pre/early pubescent stage but even more pronounced in the mid- and late pubertal stages, was remarkable and indicates—because LBM was similar in the trained and untrained groups—LBM-independent training adjustments of the O_2_ supply, likely via an increased stroke volume, as mentioned above.

In the pre/early pubertal stage, there is merely a tendency toward an expansion of the BV, and the hypothesized higher SV should be obtained by functional improvements within the circulation. In the mid- and late pubertal stages, however, the increase in BV of ∼400–600 ml in both sexes should lead to an increase in the maximum SV of ∼12 ml (Schierbauer et al., 2021). This increase, in turn, should lead to a higher maximum cardiac output (Q_max_) of ∼2.3 L** ** min^−1^ and thus a higher VO_2_max of ∼200 ml** ** min^−1^ (Schierbauer et al., 2021). Conversely, although this adjustment indicates an important improvement, it can only explain part of the difference in VO_2_max between the trained and untrained groups. In the later stages of development, both the functional improvements as in the pre/early pubertal stage and the BV increase-related optimizations should be mainly responsible for the improved VO_2_max.

A similar conclusion emerges when one examines the relationship between Hbmass and VO_2_max, as demonstrated in Figure 2. With the same Hbmass of 13.1 g** **kg LBM^−1^ (average value of boys in the mid pubertal stage), untrained children had a VO_2_max of 49.1 ml** ** min^−1**.**^ kg LBM^−1^, but trained children had a VO_2_max of 61.3 ml/min/kg LBM^−1^, indicating 25% more effectiveness in O_2_ transport and/or O_2_ metabolism. Since the trained groups of both sexes in the mid- and late pubertal stages also possess ∼1.5 g** **kg LBM^−1^ more Hbmass (see Figure 1B), their VO_2_max increases by an additional 3.5 ml** ** min^−1**.**^ kg LBM^−1^, i.e., by 7% more. The adaptation processes via increased erythropoiesis thus constitute ∼22% of the total training adaptations.

### VO_2_max and Altitude

Immediately after ascent to a moderate altitude, VO_2_max decreases by ∼0.65% per 100 m, i.e., by 17% from sea level to 2,600 m (Clark et al., 2007). During longer stays in altitude training camps at ∼2,300 m, the VO_2_max improves, but after 3 weeks, it is still ∼5% less than at sea level (Schuler et al., 2007), and the performance, measured as competition pace, is still decreased by ∼3% (Chapman and Levine, 2007). In the case of chronically altitude-adapted cyclists at the national level from Bogotá, however, comparable values (69 ml** **kg^−1**.**^ min^−1^) were measured at moderate altitudes to those of European cyclists with the same performance level at sea level (68 ml** **kg^−1**.**^ min^−1^) (Schmidt et al., 2002). To date, however, there are no data available comparing adolescent athletes born and living at different altitudes.

In the present study, we did not detect any negative, but even a tendency to a positive effect of altitude on VO_2_max (Figure 3). This result cannot be attributed to methodological causes since, when comparing the cycle ergometer and treadmill tests, the discipline-specific performance determined as power and speed at 2,600 m did not differ from that at 1,000 m. Rather, the following mechanisms could contribute to the complete compensation for the hypoxic surroundings at 2,600 m. 1) There is an increase in Hbmass, which is ∼7% in all stages of development; 2) The Hb-O_2_ binding properties are adapted to the altitude in the form of a steeper Hb-O_2_ binding curve, resulting in a left shift in the upper part and a right shift in the lower part (Schmidt et al., 1990). This process enables a higher arterial O_2_ saturation in the lungs and a more effective O_2_ release in the muscle tissue. In that study, we were able to show that, with a capillary pO_2_ of 15 mm Hg, this mechanism delivers ∼4% more O_2_ to the tissue. 3) Adequate ventilation and improved diffusion properties in the lungs (Wagner et al., 2002) lead to higher arterial O_2_ saturation at rest (Böning et al., 2004), especially during exercise, which is much more pronounced than in altitude-adapted lowlanders (Favier et al., 1995).

### Practical Importance

Based on these considerations, the importance of a high Hbmass for VO_2_max can be emphasized. Since the effects of the training-related increase in Hbmass are almost nonexistent at pre/early pubescent ages and relatively small at mid- and late pubertal ages, genetic predispositions for a high Hbmass and thus high VO_2_max in adulthood are absolute prerequisites. Screening Hbmass as one biological marker at a young age was therefore recommended by Wehrlin and Steiner, (2021) to characterize possible talent for endurance sports. In this study, for example, after correction for the training and altitude effects, 9% of all of the values of the children and adolescents from the untrained groups were greater than the 90th percentile, and 3% were greater than the 95th percentile of the sex- and puberty stage-specific Hbmass values, while the numbers for the trained children were 12 and 5%, respectively. This outcome indicates that a large number of children who have not yet practiced any sports might still have the potential to become successful endurance athletes. These subjects could be those individuals who respond to endurance training with particularly large increases in VO_2_max, as described by Bouchard et al., (1999) in their genetic studies.

Living at a moderate altitude from childhood fully compensates for the decreased ambient oxygen pressure so that VO_2_max is not restricted. Athletes from moderate altitudes, therefore, have a clear advantage in competitions at altitude over athletes from sea level even if the latter have adapted to high-altitude training camps. This advantage should also exist at lower altitudes and could be one reason for the success of elite Colombian cyclists, who mainly grew up in the area around Bogotá that we examined in this study. However, it is still unclear to what extent and in what time frame this advantage over athletes from sea level will be reduced over time when competing at low altitudes.

### Limitations

The study was conducted in Colombia, whose residents are of different ethnic origins. Although the ethnic groups in the two regions from which the children studied here derived are fairly homogeneous, it must be noted that the majority of residents of these regions are mestizos with more or less European or Native American origin. In order to be able to assess possible ethnic influences on the parameters determined here, it would therefore be desirable to carry out a genetic typing, which, however, could not be done in this study.

In the present cross-sectional study, relationships between Hbmass or BV and important confounding factors were derived by means of regression analysis. Highly significant correlations with Hbmass and other influencing factors were also established for VO_2_max. However, the correlations calculated here do not prove cause and effect but only associations between the respective variables. Cause and effect can only be estimated through longitudinal studies over several years. However, since it is extremely difficult to observe training effects from the untrained state to the competitive athlete over the course of child and adolescent development on a statistically sufficiently large number of test subjects, cross-sectional studies such as these could be valuable. Therefore, it cannot be excluded that children with a genetically predetermined high Hbmass and BV facilitating a higher VO_2_max are more likely to be found in the trained group and thus influence the conclusions about the trainability of Hbmass and BV.

In boys, the parameters related to body mass and LBM agree very well. In the case of girls, however, the data diverge notably over the course of puberty due to the increasing body fat percentage. Therefore, the hematological variables are presented as normalized to LBM to better compare boys and girls. The LBM was not measured directly here but was estimated by skin fold measurements, and based on these measurements, the body fat percentage was determined. Although this method correlates sufficiently with the gold standard methods [dual-energy X-ray absorptiometry (Hofsteenge et al., 2015), three-component method (Aguirre et al., 2015)], the results should be interpreted with caution.

The VO_2_max of the runners was corrected by 7% because it was determined on the treadmill and not on the bicycle ergometer, as was the case with the other participants. This adjustment was necessary because very well-trained cyclists show almost identical values on both devices, but runners are significantly less efficient on the ergometer. Since there are interindividual differences when comparing the VO_2_max on the treadmill and on the bicycle ergometer, inaccuracies due to the 7% correction cannot be excluded. However, since the same number of runners were tested at both altitudes, this possible inaccuracy should not affect the validity of this work.

## Conclusions

The present cross-sectional study showed, on the basis of data from 475 children and adolescents, that increases in Hbmass and BV through endurance training do not yet occur before puberty and that they are relatively moderate during and after puberty, being ∼10–12%. The large differences in Hbmass and BV in adulthood between elite athletes and untrained subjects are therefore likely to be due to genetic causes, although the underlying mechanisms are still largely unclear. Growing up at a moderate altitude, however, leads to moderate stimulation of Hbmass, being ∼7% in all stages of development.

The VO_2_max does not differ in children and adolescents growing up at different altitudes when tested in their respective places of residence; the hypoxic circumstances at moderate altitudes are completely compensated for. The training effects on VO_2_max are already clearly present before puberty and are reinforced by the onset of puberty, partly due to hematological adaptations.

## Data Availability

The datasets presented in this article are not readily available because the data are still needed for more extensive analysis. Requests to access the datasets should be directed to WS, walter.schmidt@uni-bayreuth.
