# Supplementary material for: Hemoglobin Mass, Blood Volume and VO2max of Trained and Untrained Children and Adolescents Living at Different Altitudes
Source: Front Physiol. 2022 Jun 3;13:892247. doi: 10.3389/fphys.2022.892247 (PMC9204197; doi:10.3389/fphys.2022.892247)
Supplement: Supplementary file 1 [file Table4.pdf]

Table 4: VO_2max in trained and untrained boys and girls in different stages of sexual maturation. The data is presented as absolute values as well as normalized to body mass and lean body mass (LBM). Significance of differences between boys and girls of the identical stage of maturation: * = p < 0.05, ** = p < 0-01, *** = p < 0.001. Significance of difference from the previous stage of maturation: $^{+}$ = p < 0.05, $^{++}$ = p < 0-01, $^{+++}$ = p < 0.001.

			Tanner I	Tanner II	Tanner III	Tanner IV	Tanner V	ANOVA p ≤ (Tanner, Sex, Training, Interaction)
number of boys / girls		untrained	13/11	29/21	13/17	21/14	7/6	
		trained	9/21	20/19	32/37	70/21	17/6	
VO_2max	untrained	boys	1486 ±189	1593 ±495	2141 ±395^{+++}	2310 ±400	2699 ±483	T 0.000
(ml/min)		girls	1233 ±195	1434 ±403	1624 ±350	1749 ±251	1607 ±392	S 0.000
			**		***	***	***	Tr 0.000
	trained	boys	1721 ±432	2098 ±468	2900 ±521^{+++}	3404 ±474^{+++}	3633 ±375	T x S 0.000
		girls	1510 ±240	1826 ±337^{++}	2296 ±290^{+++}	2328 ±399	2371 ±351	T x Tr 0.000
		d_{cohen}		*	***	***	***	
VO_2max	untrained	boys	47.2 ±8.1	42.2 ±12.1	42.9 ±4.7	41.2 ±6.87	45.9 ±3.6	T n.s.
(ml/min/kg)		girls	40.1 ±5.0	39.1 ±7.2	33.7 ±5.6^{+}	32.7 ±4.0	30.1 ±2.3	S 0.000
		d_{cohen}	*		***	***	***	Tr 0.000
	trained	boys	55.2 ±8.3	56.1 ±6.9	61.0 ±8.1^{+}	60.1 ±6.3	62.4 ±5.6	T x S 0.000
		girls	51.5 ±8.2	47.6 ±8.4	48.9 ±5.1	47.4 ±6.1	45.4 ±4.9	T x Tr 0.01
		d_{cohen}		**	***	***	***	
VO_2max	untrained	boys	56.8 ±7.2	51.7 ±13.1	52.5 ±7.2	48.8 ±7.3	52.6 ±3.6	T n.s.
(ml/min/kgLBM)		girls	48.8 ±5.7	49.0 ±8.9	43.4 ±7.1	43.0 ±3.5	41.5 ±4.6	S 0.000
		d_{cohen}	**		**	**	***	Tr 0.000
	trained	boys	65.7 ±10.7	66.2 ±7.9	69.9 ±7.7	68.5 ±6.3	70.1 ±6.2	T x S n.s.
		girls	61.4 ±8.4	58.2 ±9.0	60.2 ±5.8	57.9 ±6.3	58.9 ±7.1	T x Tr 0.05

		d_{cohen}		**	***	***	***	
