# Supplementary material for: Hemoglobin Mass, Blood Volume and VO2max of Trained and Untrained Children and Adolescents Living at Different Altitudes
Source: Front Physiol. 2022 Jun 3;13:892247. doi: 10.3389/fphys.2022.892247 (PMC9204197; doi:10.3389/fphys.2022.892247)
Supplement: Supplementary file 2 [file Table1.pdf]

Supplemental Material

Table 1: Methodical differentiation according to age, sex, training status, and sports discipline. - Scheme of the power increase during the VO_2max test.

	age	sex	Ergometer	initial speed, slope (treadmill)	Initial load (cycle ergomert)	Increase workload
athletes	< 14yrs	boys and girls	treadmill	5 km/h, 1°		0.5 km/h
	> 14yrs	boys		9 km/h, 1°		0.5 km/h
	> 14yrs	girls		8 km/h, 1°		0.5 km/h
cyclists speed skater race walker triathletes	< 14yrs	boys and girls	cycle ergometer		30 W	5 W
	> 14yrs	boys			cyclists 100W others 70W	15 W
	> 14yrs	girls			cyclists 70W others 50W	10 W
untrained	< 14yrs	boys and girls	cycle ergometer		20 W	5 W
	> 14yrs	boys			30 W	10 W
	> 14yrs	girls			30 W	5 W
