# Supplementary material for: Hemoglobin Mass, Blood Volume and VO2max of Trained and Untrained Children and Adolescents Living at Different Altitudes
Source: Front Physiol. 2022 Jun 3;13:892247. doi: 10.3389/fphys.2022.892247 (PMC9204197; doi:10.3389/fphys.2022.892247)
Supplement: Supplementary file 3 [file Table2.pdf]

Table 2: Anthropometric data (A) and training history (B). Values are means ±SD. The right column presents the results of the two-way ANOVA (step 1 in the statistics section). Significance of differences between boys and girls in the same stage of maturation (t-test): * = p < 0.05, ** = p < 0.01, *** = p < 0.001. Significance of differences from the previous stage of maturation (Bonferroni test): $^{+}$ = p < 0.05, $^{+++}$ = p < 0.001.

**A**		Tanner I	Tanner II	Tanner III	Tanner IV	Tanner V	ANOVA p ≤ (Tanner, Sex, Interaction)
number of boys / girls		24/37	52/48	51 / 72	104/48	27 / 12	
age (years)	boys	9.7 ±1.0	11.2 ±1.2^{+++}	13.8 ±1.8^{+++}	15.7 ±1.5^{+++}	16.5 ±1.3	T 0.000<br>S n.s.<br>I n.s.
	girls	9.9 ±1.4	10.9 ±1.2^{+}	13.8 ±1.8^{+++}	15.3 ±1.7^{+++}	15.9 ±1.6	
body mass (kg)	boys	31.6 ±4.6	38.4 ±8.7^{++}	48.2 ±8.4^{+++}	56.9 ±7.1^{+++}	58.8 ±6.6	T 0.000<br>S 0.000<br>I n.s.
	girls	30.0 ±5.3	37.6 ±8.4^{+++}	47.7 ±6.9^{+++}	52.1 ±7.5^{++}	52.5 ±7.3	
					***	*	
height (cm)	boys	134.7 ±6.9	145.3 ±8.5^{+++}	159.0 ±8.8^{+++}	167.7 ±6.9^{+++}	170.6 ±7.8	T 0.000<br>S 0.000<br>I 0.000
	girls	134.7 ±6.0	144.1 ±9.1^{+++}	155.7 ±6.3^{+++}	159.1 ±8.2	160.6 ±6.8	
				*	***	***	
BMI	boys	17.4 ±2.0	18.2 ±2.7	18.9 ±2.0	20.2 ±2.1^{++}	20.2 ±1.4	T 0.000<br>S n.s.<br>I n.s.
	girls	16.4 ±2.0	17.9 ±2.6^{+}	19.6 ±2.1^{++}	20.6 ±2.6	20.3 ±2.2	
body fat (%)	boys	16.7 ±3.7	17.2 ±6.8	14.2 ±5.3	13.1 ±4.8	11.4 ±3.1	T n.s.<br>S 0.000<br>I 0.000
	girls	16.2 ±6.7	19.0 ±5.7	20.0 ±5.6	21.3 ±6.7	24.9 ±4.6	
				***	***	***	
LBM (kg)	boys	26.3 ±3.4	31.5 ±6.0^{++}	41.2 ±6.9^{+++}	49.4 ±5.9^{+++}	52.1 ±6.1	T 0.000<br>S 0.000<br>I 0.001
	girls	24.9 ±3.6	30.1 ±5.7^{+++}	38.0 ±4.9^{+++}	40.8 ±4.8^{+}	39.2 ±4.5	
				**	***	***	

**B**							
number of boys / girls		11/25	21/25	36/53	80/27	20/6	
training volume (h/week)	boys<br>girls	11.1 ±3.7<br>11.4 ±4.0	10.3 ±4.0<br>12.2 ±3.3	14.1 ±5.0<br>14.4 ±5.5	16.2 ±5.9<br>15.1 ±7.2	19.2 ±8.1<br>19.0 ±4.5	T 0.000<br>S n.s.<br>I n.s.
training history (years)	boys<br>girls	2.8 ±1.9<br>2.9 ±1.9	2.2 ±1.8<br>3.1 ±2.3	3.2 ±2.0<br>4.0 ±2.4	3.9 ±2.8<br>4.7 ±2.4	4.4 ±2.5<br>6.2 ±4.7	T 0.000<br>S 0.05<br>I n.s.
