# Supplementary material for: Hemoglobin Mass, Blood Volume and VO2max of Trained and Untrained Children and Adolescents Living at Different Altitudes
Source: Front Physiol. 2022 Jun 3;13:892247. doi: 10.3389/fphys.2022.892247 (PMC9204197; doi:10.3389/fphys.2022.892247)
Supplement: Supplementary file 4 [file Table3.pdf]

Table 3: Hematological data and blood volumes in boys and girls in different stages of sexual maturation. The data is presented as absolute values as well as normalized to body mass and lean body mass (LBM). Hbmass = hemoglobin mass, RCV = red cell volume, BV = blood volume, PV = plasma volume, Significance of differences between boys and girls of the identical stage of maturation: * = p < 0.05, ** = p < 0-01, *** = p < 0.001. Significance of difference from the previous stage of maturation: $^{+}$ = p < 0.05, $^{++}$ = p < 0-01, $^{+++}$ = p < 0.001.

		Tanner I	Tanner II	Tanner III	Tanner IV	Tanner V	ANOVA p ≤ (Tanner, Sex, Interaction)
number of boys / girls		24/37	52/48	51/72	104/48	27/12	
[Hb]	boys	14.6 ±1.0	14.5 ±0.9	15.1 ±1.2^{+}	15.8 ±1.2^{+++}	15.8 ±1.4	T 0.000
(g/dl)	girls	14.4 ±0.9	14.2 ±0.8	14.2 ±1.0	14.2 ±1.1	14.3 ±1.1	S 0.000
	d_{cohen}		*	***	***	***	I 0.000
Hct	boys	42.1 ±2.5	42.0 ±2.3;	43.9 ±2.9^{+++}	46.4 ±3.0^{+++}	46.7 ±3.0	T 0.000
(%)	girls	41.8 ±24	41.8 ±2.2;	42.2 ±2.6	42.2 ±3.1	42.4 ±2.8	S 0.000
				***	***	***	I 0.000
Hbmass	boys	364 ±56	429 ±92	636 ±152^{+++}	775 ±134^{+++}	839 ±120	T 0.000
(g)	girls	326 ±61	399 ±89^{++}	503 ±104^{+++}	539 ±112	498 ±77	S 0.000
	d_{cohen}	*		***	***	***	I 0.000
Hbmass	boys	11.6 ±1.4	11.3 ±1.6	13.1 ±1.8^{+++}	13.6 ±1.5	14.3 ±1.1	T 0.000
(g/kg)	girls	11.0 ±1.6	10.7 ±1.2	10.6 ±1.7	10.3 ±1.4	9.5 ±1.0	S 0.000
	d_{cohen}			***	***	***	I 0.000
Hbmass	boys	13.9 ±1.5	13.6 ±1.7	15.3 ±1.7^{+++}	15.6 ±1.5	16.1 ±1.2	T 0.000
(g/kgLBM)	girls	13.1 ±1.8	13.2 ±1.5	13.2 ±1.9	13.1 ±1.6	12.6 ±0.9	S 0.000
	d_{cohen}			***	***	***	I 0.000
RCV	boys	1054 ±175	1241 ±273	1849 ±452^{+++}	2276 ±392^{+++}	2490 ±385	T 0.000
(ml)	girls	950 ±181	1179 ±267^{++}	1490 ±307^{+++}	1607 ±341	1479 ±240	S 0.000

	d_{cohen}	*		***	***	***	I 0.000
RCV<br>(ml/kg)	boys<br>girls<br>d_{cohen}	33.5 ±4.1<br>31.9 ±4.7	32.3 ±4.7<br>31.5 ±3.7	38.2 ±5.2^{+++}<br>31.3 ±5.0<br>***	40.0 ±4.6<br>30.8 ±4.4<br>***	42.2 ±3.6<br>28.2 ±3.1<br>***	T 0.000<br>S 0.000<br>I 0.000
RCV<br>(ml/kgLBM)	boys<br>girls<br>d_{cohen}	40.0 ±4.4<br>38.3 ±5.3	39.0 ±4.7<br>39.1 ±4.5	44.5 ±5.1^{+++}<br>39.1 ±5.6<br>***	45.9 ±4.3<br>39.2 ±5.2<br>***	47.7 ±3.6<br>37.5 ±2.8<br>***	T 0.000<br>S 0.000<br>I 0.000
BV<br>(ml)	boys<br>girls<br>d_{cohen}	2756±440<br>2497 ±470<br>*	3246 ±691<br>3097 ±665^{++}	4622 ±1050^{+++}<br>3883 ±756^{+++}<br>***	5397 ±872^{+++}<br>4179 ±789<br>***	5873 ±1062^{+}<br>3847 ±648<br>***	T 0.000<br>S 0.000<br>I 0.000
BV<br>(ml/kg)	boys<br>girls<br>d_{cohen}	87.6 ±10.2<br>84.0 ±13.2	84.4 ±10.4<br>83.1 ±10.1	95.6 ±11.4^{+++}<br>81.6 ±11.9<br>***	94.9 ±9.8<br>80.2 ±9.9<br>***	99.5 ±10.9<br>73.6 ±10.2<br>***	T 0.05<br>S 0.000<br>I 0.000
BV<br>(ml/kgLBM)	boys<br>girls<br>d_{cohen}	104.7 ±10.8<br>100.9 ±14.8	102.1 ±10.8<br>102.8 ±11.5	111.6 ±12.4^{+++}<br>102.0 ±13.3<br>***	109.0 ±9.1<br>102.2 ±12.6<br>***	112.1 ±10.6<br>97.7 ±9.6<br>***	T 0.05<br>S 0.000<br>I 0.01
PV<br>(ml)	boys<br>girls<br>d_{cohen}	1702 ±282<br>1547 ±302<br>*	2005 ±432<br>1917 ±410^{++}	2772 ±629^{+++}<br>2393 ±473^{+++}<br>***	3121 ±529^{+++}<br>2572 ±485<br>***	3383 ±723<br>2367 ±434<br>***	T 0.000<br>S 0.000<br>I 0.000
PV<br>(ml/kg)	boys<br>girls<br>d_{cohen}	54.1 ±7.0<br>52.1 ±9.0	52.1 ±6.2<br>51.5 ±7.1	57.4 ±7.3^{++}<br>50.3 ±7.5<br>***	54.9 ±6.5<br>49.4 ±6.5<br>***	57.2 ±8.4<br>45.3 ±7.5<br>***	T n.s.<br>S 0.000<br>I 0.000
PV<br>(ml/kgLBM)	boys<br>girls<br>d_{cohen}	65.0 ±7.4<br>62.5 ±10.1	63.0 ±6.9;<br>63.7 ±7.9	67.1 ±8.7^{+}<br>62.8 ±8.6<br>**	63.1 ±6.6^{+}<br>63.0 ±8.8	64.5 ±8.4<br>60.2 ±7.7	T n.s.<br>S 0.05<br>I n.s.
